# Early Identification of Autism Spectrum Disorder Among Children Aged 4 Years — Early Autism and Developmental Disabilities Monitoring Network, Six Sites, United States, 2016

**DOI:** 10.15585/mmwr.ss6903a1

**Published:** 2020-03-27

**Authors:** Kelly A. Shaw, Matthew J. Maenner, Jon Baio, EdS, Anita Washington, Deborah L. Christensen, Lisa D. Wiggins, Sydney Pettygrove, Jennifer G. Andrews, Tiffany White, Cordelia Robinson Rosenberg, John N. Constantino, Robert T. Fitzgerald, Walter Zahorodny, Josephine Shenouda, Julie L. Daniels, Angelica Salinas, Maureen S. Durkin, Patricia M. Dietz

**Affiliations:** ^1^National Center on Birth Defects and Developmental Disabilities, CDC, Atlanta, Georgia; ^2^University of Arizona, Tucson; ^3^Colorado Department of Public Health and Environment, Denver; ^4^University of Colorado School of Medicine, Department of Pediatrics, Aurora; ^5^Washington University in St. Louis, Missouri; ^6^Rutgers New Jersey Medical School, Newark; ^7^University of North Carolina, Chapel Hill; ^8^University of Wisconsin, Madison

## Abstract

**Problem/Condition:**

Autism spectrum disorder (ASD).

**Period Covered:**

2016.

**Description of System:**

The Early Autism and Developmental Disabilities Monitoring (Early ADDM) Network, a subset of the overall ADDM Network, is an active surveillance program that estimates ASD prevalence and monitors early identification of ASD among children aged 4 years. Children included in surveillance year 2016 were born in 2012 and had a parent or guardian who lived in the surveillance area in Arizona, Colorado, Missouri, New Jersey, North Carolina, or Wisconsin, at any time during 2016. Children were identified from records of community sources including general pediatric health clinics, special education programs, and early intervention programs. Data from comprehensive evaluations performed by community professionals were abstracted and reviewed by trained clinicians using a standardized ASD surveillance case definition with criteria from the *Diagnostic and Statistical Manual of Mental Disorders, Fifth Edition* (DSM-5).

**Results:**

In 2016, the overall ASD prevalence was 15.6 per 1,000 (one in 64) children aged 4 years for Early ADDM Network sites. Prevalence varied from 8.8 per 1,000 in Missouri to 25.3 per 1,000 in New Jersey. At every site, prevalence was higher among boys than among girls, with an overall male-to-female prevalence ratio of 3.5 (95% confidence interval [CI] = 3.1–4.1). Prevalence of ASD between non-Hispanic white (white) and non-Hispanic black (black) children was similar at each site (overall prevalence ratio: 0.9; 95% CI = 0.8–1.1). The prevalence of ASD using DSM-5 criteria was lower than the prevalence using *Diagnostic and Statistical Manual of Mental Disorders, Fourth Edition, Text Revision* (DSM-IV-TR) criteria at one of four sites that used criteria from both editions. Among sites where ≥60% of children aged 4 years had information about intellectual disability (intelligence quotient ≤70 or examiner’s statement of intellectual disability documented in an evaluation), 53% of children with ASD had co-occurring intellectual disability. Of all children aged 4 years with ASD, 84% had a first evaluation at age ≤36 months and 71% of children who met the surveillance case definition had a previous ASD diagnosis from a community provider. Median age at first evaluation and diagnosis for this age group was 26 months and 33 months, respectively. Cumulative incidence of autism diagnoses received by age 48 months was higher for children aged 4 years than for those aged 8 years identified in Early ADDM Network surveillance areas in 2016.

**Interpretation:**

In 2016, the overall prevalence of ASD in the Early ADDM Network using DSM-5 criteria (15.6 per 1,000 children aged 4 years) was higher than the 2014 estimate using DSM-5 criteria (14.1 per 1,000). Children born in 2012 had a higher cumulative incidence of ASD diagnoses by age 48 months compared with children born in 2008, which indicates more early identification of ASD in the younger group. The disparity in ASD prevalence has decreased between white and black children. Prevalence of co-occurring intellectual disability was higher than in 2014, suggesting children with intellectual disability continue to be identified at younger ages. More children received evaluations by age 36 months in 2016 than in 2014, which is consistent with *Healthy People 2020* goals. Median age at earliest ASD diagnosis has not changed considerably since 2014.

**Public Health Action:**

More children aged 4 years with ASD are being evaluated by age 36 months and diagnosed by age 48 months, but there is still room for improvement in early identification. Timely evaluation of children by community providers as soon as developmental concerns have been identified might result in earlier ASD diagnoses, earlier receipt of evidence-based interventions, and improved developmental outcomes.

## Introduction

Autism spectrum disorder (ASD) is a developmental disability characterized by deficits in social interaction, restricted interests, and repetitive behaviors. In 2000, CDC began active population-based surveillance of ASD prevalence among children aged 8 years at multiple U.S. sites participating in the Autism and Developmental Disabilities Monitoring (ADDM) Network (*1*–*8*). In 2016, the overall prevalence of ASD among children aged 8 years in the ADDM Network was 18.5 per 1,000 (one in 54) children (*8*) on the basis of *Diagnostic and Statistical Manual of Mental Disorders, Fifth Edition* (DSM-5) criteria (*9*). Although study areas and methods of ascertainment are not completely comparable, this estimate is 175% higher than the first estimates reported by the ADDM Network in 2000 and 2002.

Since 2010, a subset of ADDM Network sites known as the Early ADDM Network also has collected data on ASD prevalence and early identification metrics among children aged 4 years. Similar to children aged 8 years, ASD prevalence for those children has been higher over time, from 13.4 per 1,000 children aged 4 years in 2010 to 17.0 per 1,000 children aged 4 years in 2014 (*10*,*11*) using *Diagnostic and Statistical Manual of Mental Disorders, Fourth Edition, Text Revision* (DSM-IV-TR) criteria (*12*). In 2014, Early ADDM Network sites also used DSM-5 criteria to classify cases; reported overall ASD prevalence with the updated criteria was 14.1 per 1,000 children aged 4 years. Whereas sites have identified higher ASD prevalence among children aged 4 years over time, prevalence among this group has been consistently lower than among children aged 8 years.

Groups such as the American Academy of Pediatrics encourage early routine screening for ASD and other developmental concerns (*13*) because early diagnosis (i.e., as soon as possible after concerns have been noticed) could lead to intervention that can improve developmental outcomes (*14*–*17*). In addition to estimating prevalence and monitoring trends in early identification of ASD through the Early ADDM Network, CDC also provides information and resources for monitoring a child’s development through the “Learn the Signs. Act Early.” program. One such resource is the Milestone Tracker app (https://www.cdc.gov/ncbddd/actearly).

This report presents the estimated prevalence of ASD and co-occurring intellectual disability (intelligence quotient [IQ] ≤70 or an examiner’s statement of intellectual disability documented in an evaluation) among children aged 4 years identified by Early ADDM Network sites in 2016. This report also includes information about early identification of ASD, including median age at first evaluation and diagnosis and cumulative incidence of ASD diagnoses among children aged 4 and 8 years in Early ADDM Network surveillance areas. These data can be used by persons working in health care, education, policy, or research settings to monitor trends in prevalence and early identification and provide insight into future service needs so children with ASD can receive evidence-based interventions as early as possible.

## Methods

To estimate the prevalence of ASD among children aged 4 years, six of the 11 ADDM Network sites conducting surveillance among children aged 8 years also collected surveillance data for children aged 4 years as part of the Early ADDM Network. Surveillance among children aged 4 years was conducted in a subset of the surveillance area for children aged 8 years at all sites except North Carolina, where surveillance areas were the same. Children included in surveillance year 2016 analysis were born in 2012 and had a parent or guardian who lived in an Early ADDM Network surveillance area in Arizona, Colorado, Missouri, New Jersey, North Carolina, or Wisconsin, at any time during 2016 (Table 1). All sites functioned as public health authorities under the Health Insurance Portability and Accountability Act of 1996 Privacy Rule and met applicable local institutional review board, privacy, and confidentiality requirements under 45 CFR 46 (*18*).

**TABLE 1:** Prevalence* of autism spectrum disorder among children aged 4 years, overall and by sex — Early Autism and Developmental Disabilities Monitoring Network, six sites, United States, 2016

Site	Description of surveillance area	Overall			Male	Female	Male-to-female prevalence ratio (95% CI)^¶^
		Total population^†^	No. with ASD	Prevalence (95% CI)^§^	Prevalence (95% CI)	Prevalence (95% CI)	
Arizona	Part of one county in metropolitan Phoenix**	8,997	96	10.7 (8.7–13.0)	15.8 (12.6–19.8)	5.1 (3.4– 7.7)	3.1 (1.9–4.9)
Colorado	One county in metropolitan Denver	8,082	116	14.4 (12.0–17.2)	22.3 (18.2–27.3)	6.1 (4.1– 9.0)	3.7 (2.4–5.7)
Missouri	One county in metropolitan St. Louis	11,965	105	8.8 (7.3–10.6)	13.4 (10.8–16.5)	3.9 (2.6– 5.9)	3.4 (2.1–5.3)
New Jersey	One full county and part of one county that includes metropolitan Newark**	17,633	446	25.3 (23.1–27.7)	38.7 (35.0–42.9)	11.0 (9.0–13.4)	3.5 (2.8–4.4)
North Carolina	Four counties in central North Carolina	17,545	207	11.8 (10.3–13.5)	19.4 (16.7–22.5)	3.9 (2.8– 5.5)	4.9 (3.4–7.1)
Wisconsin	Two counties in south central Wisconsin	8,055	155	19.2 (16.5–22.5)	27.8 (23.2–33.3)	10.5 (7.8–14.2)	2.6 (1.9–3.7)
**Total**		**72,277**	**1,125**	**15.6 (14.7–16.5)**	**23.9 (22.4–25.5)**	**6.8 (6.0– 7.7)**	**3.5 (3.1–4.1)**

**Abbreviations:** ASD = autism spectrum disorder; CI = confidence interval.

* Per 1,000 children aged 4 years.

^†^ Total numbers of children aged 4 years in each surveillance area were obtained from CDC’s National Center for Health Statistics vintage 2018 bridged-race population estimates for 2016 (https://www.cdc.gov/nchs).

^§^ CIs were calculated using the Wilson score method.

^¶^ Wilson score 95% CIs exclude 1.0, indicating higher prevalence among males than females in all sites.

** Denominator excludes school districts that were not included in the surveillance area using National Center for Education Statistics kindergarten population estimates for 2016 (as a proxy for unavailable 2017 kindergarten estimates; https://nces.ed.gov/ccd/pubagency.asp).

### Case Ascertainment and Surveillance Case Definition

The Early ADDM Network used the same two-phase surveillance methodology as the ADDM Network, which has been described previously (*7*,*11*). In the first phase, ADDM Network staff reviewed records with selected billing codes from the *International Classification of Disease, Ninth Revision* (ICD-9) *or International Classification of Diseases, Tenth Revision* (ICD-10), special education exceptionalities, or early intervention program eligibility classifications. A list of qualifying codes and exceptionalities is available (Supplementary Table 1, https://stacks.cdc.gov/view/cdc/85336). If any record contained a statement of suspicion of ASD or description of behavior consistent with ASD, information (e.g., demographics and evaluations) from all available community sources was abstracted into a composite record. All sites reviewed records from health care sources. Special education records (for children aged ≥3 years) were reviewed in Arizona, Colorado, and New Jersey and three of four counties in North Carolina. Early intervention program records (for children aged <3 years) were reviewed in New Jersey, North Carolina, and Wisconsin.

In the second phase, ADDM Network clinicians reviewed the compiled, deidentified records to determine case status using DSM-5 criteria. In Arizona, Colorado, Missouri, and New Jersey, ≥50% of records also were reviewed using previous DSM-IV-TR criteria. DSM-5 and DSM-IV-TR criteria were described previously (*7*). The ASD surveillance case definition was met if behaviors described in records were consistent with diagnostic criteria or if an ASD diagnosis was documented in an evaluation. A clinician reviewer could rule out ASD if information in the record was conflicting or insufficient or if other diagnosed conditions better accounted for a child’s symptoms. A blinded, random sample of 10% of abstracted records was scored independently by a second clinician reviewer to assess interrater reliability for 2016 (Cohen’s kappa = 0.92).

### Additional Data Sources and Variable Definitions

The total number of children aged 4 years living in each surveillance area was obtained from the National Center for Health Statistics (NCHS) vintage 2018 postcensal bridged-race population estimates for 2016 (https://www.cdc.gov/nchs). In Arizona and Utah, the surveillance area included a subset of school districts in two counties. NCHS race and ethnicity and sex category estimates for those counties were adjusted by the proportion of children in each race and ethnicity and sex category in the included districts compared with all school districts in the county. For school district population estimates, National Center for Education Statistics (NCES) Common Core of Data 2016 kindergarten population estimates were used as a proxy for unavailable 2017 kindergarten populations (https://nces.ed.gov/ccd/pubagency.asp). Because Early ADDM Network surveillance areas are a subset of the overall ADDM Network surveillance areas, denominators for prevalence and cumulative incidence of ASD among children aged 8 years living in Early ADDM Network surveillance areas also were calculated using NCHS estimates for children aged 8 years (https://www.cdc.gov/nchs) and NCES estimates for third graders (https://nces.ed.gov/ccd/pubagency.asp).

Data for age at first evaluation were restricted to children born in state (determined by a match to a birth certificate from the state where the child was identified) because out-of-state evaluations might not be available from community sources. Children were considered to have co-occurring intellectual disability if they had a score ≤70 on their most recent IQ test or an examiner’s statement of intellectual disability in a developmental evaluation. Analyses of intellectual disability were restricted to sites where ≥60% of children with ASD had information about intellectual disability.

### Analytic Methods

ASD prevalence was calculated as the number of children who met the ASD surveillance case definition divided by the number of children living in the surveillance area. Full case counts and denominators for estimates among children aged 4 years are available (Supplementary Table 2, https://stacks.cdc.gov/view/cdc/85336). Prevalence ratios were used to compare prevalence by sex, race and ethnicity, and diagnostic criteria. Race-specific prevalence estimates were limited to non-Hispanic white (white), non-Hispanic black (black), and Hispanic children because of small numbers in other race and ethnicity categories. Agreement for case classification between DSM-5 and DSM-IV-TR criteria was measured using Cohen’s kappa. Cumulative incidence and prevalence for children aged 4 years (born in 2012) were compared with the subset of children aged 8 years (born in 2008) from the ADDM Network who lived in the Early ADDM Network surveillance areas. Cumulative incidence of ASD diagnosis was calculated by dividing the total number of children with diagnoses at each month of age by the 2016 population denominator for each age group. The Wilson score method was used to calculate 95% confidence intervals (CIs). Prevalence ratios were considered significant at an alpha level of 0.05 when the 95% CIs did not include the null value of 1. Differences in medians were tested using permutation tests with a significance threshold of p<0.05. The male-to-female prevalence ratio among children aged 4 years was compared with the ratio among children aged 8 years in the overall ADDM Network using the Mantel-Haenszel test of homogeneity with a significance threshold of p<0.05. R software (version 3.5.3; R Foundation) was used for all data analysis and visualizations*.* Additional information about packages used for analysis and visualization is available (Supplementary Table 3, https://stacks.cdc.gov/view/cdc/85336).

## Results

In 2016, ASD prevalence in the six sites was 15.6 per 1,000 (one in 64) children aged 4 years overall using DSM-5 criteria. Estimates ranged from 8.8 per 1,000 in Missouri to 25.3 per 1,000 in New Jersey (Table 1). Among boys aged 4 years, prevalence was 23.9 per 1,000, 3.5 times higher than the 6.8 per 1,000 prevalence among girls aged 4 years.

Population distributions and ASD prevalence estimates by race and ethnicity varied among sites (Table 2) (Supplementary Table 2, https://stacks.cdc.gov/view/cdc/85336). Prevalence between white and black children did not differ at any site. Overall, ASD prevalence was 13.2 per 1,000 among white children aged 4 years and 14.3 per 1,000 among black children aged 4 years. Prevalence among white and black children was lower than among Hispanic children at one site (New Jersey), where the white-to-Hispanic and black-to-Hispanic prevalence ratios were both 0.6.

**TABLE 2:** Prevalence* of autism spectrum disorder among children aged 4 years, by race/ethnicity — Early Autism and Developmental Disabilities Monitoring Network, six sites, United States, 2016

Site	Non-Hispanic white	Non-Hispanic black	Hispanic	Prevalence ratios		
	Prevalence (95% CI)^†^	Prevalence (95% CI)	Prevalence (95% CI)	Non-Hispanic white to non-Hispanic black (95%CI)	Non-Hispanic white to Hispanic (95% CI)	Non-Hispanic black to Hispanic (95% CI)
Arizona	11.0 (8.5–14.3)	10.3 (4.0–26.1)	7.7 (5.2–11.5)	1.1 (0.4–2.8)	1.4 (0.9–2.3)	1.3 (0.5–3.6)
Colorado	14.4 (10.9–19.1)	11.6 (6.5–20.6)	14.3 (10.9–18.9)	1.2 (0.7–2.4)	1.0 (0.7–1.5)	0.8 (0.4–1.5)
Missouri	9.0 (7.0–11.5)	7.1 (4.9–10.2)	5.0 (1.7–14.6)	1.3 (0.8–2.0)	1.8 (0.6–5.4)	1.4 (0.5–4.4)
New Jersey	19.9 (16.3–24.2)	20.4 (17.1–24.4)	32.1 (28.0–36.7)	1.0 (0.7–1.3)	0.6 (0.5–0.8)^§^	0.6 (0.5–0.8)^§^
North Carolina	11.5 (9.5–13.8)	12.3 (9.2–16.5)	7.8 (5.3–11.4)	0.9 (0.7–1.3)	1.5 (1.0–2.3)	1.6 (1.0–2.6)
Wisconsin	16.7 (13.7–20.4)	18.7 (11.3–30.6)	23.1 (15.4–34.4)	0.9 (0.5–1.5)	0.7 (0.5–1.1)	0.8 (0.4–1.5)
**Total**	**13.2 (12.0–14.4)**	**14.3 (12.5–16.3)**	**18.4 (16.5–20.5)**	**0.9 (0.8–1.1)**	**0.7 (0.6–0.8)** ^§^	**0.8 (0.7–0.9)** ^§^

**Abbreviation:** CI = confidence interval.

* Per 1,000 children aged 4 years.

^†^ CIs were calculated using the Wilson score method.

^§^ Wilson score 95% CIs exclude 1.0, indicating lower prevalence for non-Hispanic white or non-Hispanic black compared with Hispanic children.

Using DSM-5 criteria, prevalence of ASD was lower compared with using DSM-IV-TR criteria at one site (New Jersey) of four sites that reviewed ≥50% of records using both criteria (Table 3). Overall prevalence at the four sites was 16.3 per 1,000 children aged 4 years using DSM-5 criteria and 18.9 per 1,000 children aged 4 years using DSM-IV-TR (prevalence ratio: 0.9; Cohen’s kappa = 0.83).

**TABLE 3:** Comparison of autism spectrum disorder prevalence among children aged 4 years using criteria from *Diagnostic and Statistical Manual of Mental Disorders, Fifth Edition,* and *Diagnostic and Statistical Manual of Mental Disorders, Fourth Edition, Text Revision* — Early Autism and Developmental Disabilities Monitoring Network, four sites,* United States, 2016

Site	No. of children who met case criteria				Prevalence			Cohen’s kappa
	DSM-5 and DSM-IV-TR	DSM-5 only	DSM-IV-TR only	Neither	DSM-5 (95% CI)^†^	DSM-IV-TR (95% CI)	DSM-5–to–DSM-IV-TR ratio (95% CI)	
Arizona	92	4	29	318	10.7 (8.7–13.0)	13.4 (11.3–16.0)	0.8 (0.6–1.0)	0.80
Colorado	108	8	12	69	14.4 (12.0–17.2)	14.8 (12.4–17.7)	1.0 (0.8–1.2)	0.79
Missouri	99	6	4	35	8.8 (7.3–10.6)	8.6 (7.1–10.4)	1.0 (0.8–1.3)	0.83
New Jersey	439	7	98	650	25.3 (23.1–27.7)	30.5 (28.0–33.1)	0.8 (0.7–0.9)^§^	0.82
**Total**	**738**	**25**	**143**	**1,072**	**16.3 (15.2–17.5)**	**18.9 (17.7–20.1)**	**0.9 (0.8–1.0)** ^§^	**0.83**

**Abbreviations:** CI = confidence interval; DSM-5 = *Diagnostic and Statistical Manual of Mental Disorders, Fifth Edition*; DSM-IV-TR = *Diagnostic and Statistical Manual of Mental Disorders, Fourth Edition, Text Revision*.

* Includes only sites with DSM-IV-TR review data available for ≥50% of children.

^†^ CIs were calculated using the Wilson score method.

^§^ Before rounding, Wilson score 95% CIs exclude 1.0, indicating lower prevalence using DSM-5 criteria compared with DSM-IV-TR criteria.

Four sites had intellectual disability data for at least 60% of children with ASD (Table 4). Of children with ASD, 53% had co-occurring intellectual disability. The highest percentage of children aged 4 years with ASD and intellectual disability was in North Carolina (66%) and the lowest was in Colorado (29%).

**TABLE 4:** Children aged 4 years with autism spectrum disorder and co-occurring intellectual disability,* by site^†^ and sex — Early Autism and Developmental Disabilities Monitoring Network, four sites, United States, 2016

Site/Sex	ID data available	Co-occurring ID	
	No. (%)	No.	% (95% CI)^§^
**Site**			
Arizona	77 (80.2)	34	44.2 (33.6–55.3)
Colorado	70 (60.3)	20	28.6 (19.3–40.1)
New Jersey	390 (87.4)	208	53.3 (48.4–58.2)
North Carolina	149 (72.0)	99	66.4 (58.5–73.5)
**Sex**			
Male	555 (80.3)	288	51.9 (47.7–56.0)
Female	131 (75.3)	73	55.7 (47.2–63.9)
**Total**	**686 (79.3)**	**361**	**52.6 (48.9–56.3)**

**Abbreviations:** CI = confidence interval; ID = intellectual disability.

* Intelligence quotient ≤70 or an examiner’s statement of intellectual disability documented in an evaluation.

^†^ Includes only sites with intellectual disability data available for ≥60% of children with autism spectrum disorder.

^§^ CIs were calculated using the Wilson score method.

Among children aged 4 years with ASD, 80%–84% were matched to a birth certificate from the state where they were identified (Table 5). Median age at earliest comprehensive evaluation for these children was 26 months overall (range: 22 months [North Carolina] to 30 months [Arizona and Missouri]). Across sites, 84% of children with ASD had a first evaluation at age ≤36 months.

**TABLE 5:** Ages at first evaluation for children with autism spectrum disorder matched to an in-state birth certificate,* by site and sex — Early Autism and Developmental Disabilities Monitoring Network, six sites, United States, 2016

Site/Sex	Children with ASD and a matched in-state birth certificate	First evaluation at age ≤36 mos		Median age (mos) at first evaluation
	No. (%)	No.	% (95% CI)^†^	
**Site**				
Arizona	77 (80.2)	54	70.1 (59.2–79.2)	30.0
Colorado	97 (83.6)	80	82.5 (73.7–88.8)	29.0
Missouri	85 (81.0)	57	67.1 (56.5–76.1)	30.0
New Jersey	368 (82.5)	315	85.6 (81.6–88.8)	25.0
North Carolina	170 (82.1)	159	93.5 (88.8–96.3)	22.0
Wisconsin	130 (83.9)	115	88.5 (81.8–92.9)	24.0
**Sex**				
Male	722 (81.5)	608	84.2 (81.4–86.7)	25.5
Female	205 (85.8)	172	83.9 (78.3–88.3)	26.0
**Total**	**927 (82.4)**	**780**	**84.1 (81.7–86.4)**	**26.0**

**Abbreviations:** ASD = autism spectrum disorder; CI = confidence interval.

* Limited to children with a birth certificate from the state where they were identified because out-of-state evaluations might not be available from community sources.

^†^ CIs were calculated using the Wilson score method.

Documented ASD diagnoses were found for 71% of children aged 4 years who met the ASD surveillance case definition across sites. The median age at earliest diagnosis was 33 months (Table 6). Children in North Carolina had the youngest median age at earliest diagnosis (29 months) but the lowest percentage of documented diagnoses (55%).

**TABLE 6:** Median age at earliest autism spectrum disorder diagnosis among children with documented diagnosis, by site and sex — Early Autism and Developmental Disabilities Monitoring Network, six sites, United States, 2016

Site/Sex	Children with a documented ASD diagnosis		Median age (mos) at earliest ASD diagnosis
	No.	% (95% CI)*	
**Site**			
Arizona	55	57.3 (47.3–66.7)	35.0
Colorado	72	62.1 (53.0–70.4)	32.5
Missouri	96	91.4 (84.5–95.4)	34.0
New Jersey	337	75.6 (71.4–79.3)	32.0
North Carolina	114	55.1 (48.3–61.7)	29.0
Wisconsin	124	80.0 (73.0–85.5)	36.0
**Sex**			
Male	622	70.2 (67.1–73.1)	33.0
Female	176	73.6 (67.7–78.8)	32.0
**Total**	**798**	**70.9 (68.2–73.5)**	**33.0**

**Abbreviations:** ASD = autism spectrum disorder; CI = confidence interval.

* CIs were calculated using the Wilson score method.

Across sites, children with and without intellectual disability had median ages at earliest evaluation of 25 and 24 months, respectively, and the same median age at earliest diagnosis, 32 months (Table 7). Children with missing intellectual disability data had a median age at earliest evaluation of 32 months, which was higher than for children with any intellectual disability information, and median age at earliest diagnosis of 34 months, which was not different from children with intellectual disability data.

**TABLE 7:** Median age at earliest evaluation and autism spectrum disorder diagnosis, by intellectual disability* status — Early Autism and Developmental Disabilities Monitoring Network, four sites,^ †^ United States, 2016

ID status	No.	Median age (mos) at earliest evaluation^§^	Median age (mos) at earliest diagnosis^¶^
**Arizona**			
No ID	43	29.5	36.0
ID	34	31.0	40.0
Missing	19	31.0	33.5
**Colorado**			
No ID	50	27.5	32.5
ID	20	26.0	29.5
Missing	46	34.0	35.0
**New Jersey**			
No ID	182	23.0	30.0
ID	208	26.0	33.0
Missing	56	34.0	36.0
**North Carolina**			
No ID	50	22.0	32.0
ID	99	22.0	28.0
Missing	58	24.0	30.0
**Total**			
**No ID**	**325**	**24.0**	**32.0**
**ID**	**361**	**25.0**	**32.0**
**Missing**	**179**	**32.0**	**34.0**

**Abbreviation:** ID = intellectual disability.

* Intelligence quotient ≤70 or an examiner’s statement of intellectual disability documented in an evaluation.

^†^ Includes only sites with intellectual disability data available for ≥60% of children with autism spectrum disorder.

^§^ Limited to 712 children with a birth certificate from the state where they were identified.

^¶^ Limited to 578 children with a known diagnosis.

**FIGURE 1:**
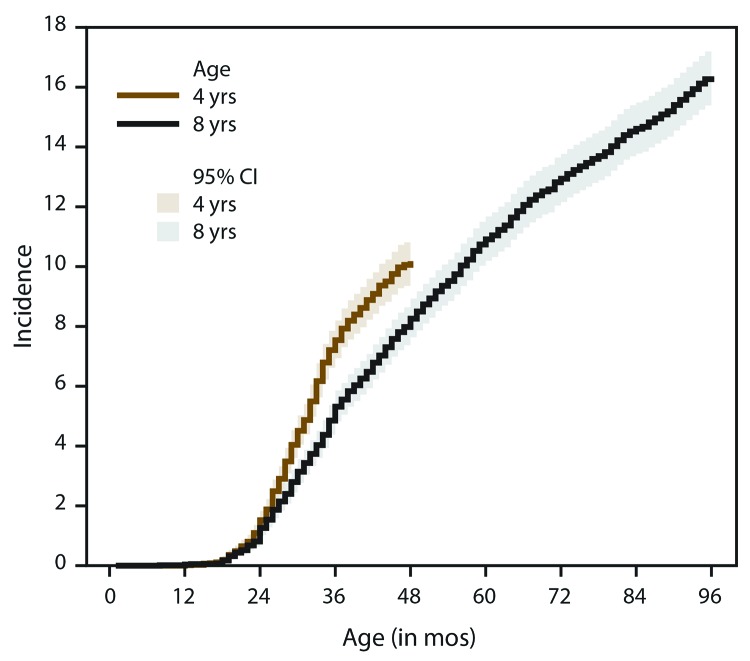
Cumulative incidence* of autism spectrum disorder diagnoses among children aged 4 or 8 years, by month of age at earliest documented diagnosis — Early Autism and Developmental Disabilities Monitoring Network, six sites,^†^ United States, 2016 **Abbreviation:** CI = confidence interval. * Per 1,000 children aged 4 or 8 years. ^†^ Arizona, Colorado, Missouri, New Jersey, North Carolina, and Wisconsin.

**FIGURE 2:**
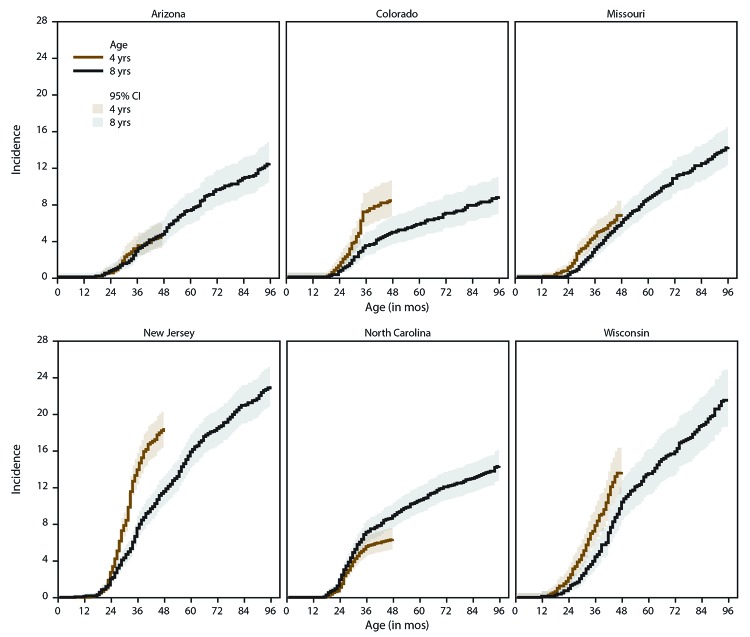
Cumulative incidence* of autism spectrum disorder diagnoses among children aged 4 or 8 years, by site and month of age at earliest documented diagnosis — Early Autism and Developmental Disabilities Monitoring Network, six sites, United States, 2016 **Abbreviation:** CI = confidence interval. * Per 1,000 children aged 4 or 8 years.

Compared with children aged 4 years, children aged 8 years in Early ADDM Network surveillance areas had a higher ASD prevalence overall at 23.5 per 1,000 children (range: 16.3 per 1,000 [Colorado] to 30.6 per 1,000 [New Jersey]) (Supplementary Table 4, https://stacks.cdc.gov/view/cdc/85336). However, the cumulative incidence of ASD diagnosis was higher for children aged 4 years than for those aged 8 years from ages 26–48 months (Figure 1). At age 48 months, cumulative incidence was 10.2 per 1,000 children aged 4 years and 8.3 per 1,000 children aged 8 years. The pattern was evident for four of six sites; however, the incidence curve for children aged 4 years overlapped with the curve for children aged 8 years in Arizona and was lower than the curve for children aged 8 years in North Carolina (Figure 2).

**FIGURE 3:**
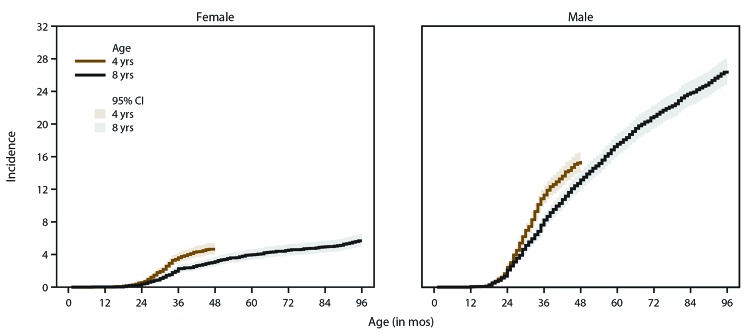
Cumulative incidence* of autism spectrum disorder diagnoses among children aged 4 or 8 years, by sex and month of age at earliest documented diagnosis — Early Autism and Developmental Disabilities Monitoring Network, six sites,^†^ United States, 2016 **Abbreviation:** CI = confidence interval. * Per 1,000 children aged 4 or 8 years. ^†^ Arizona, Colorado, Missouri, New Jersey, North Carolina, and Wisconsin.

When stratified by sex, the same pattern of higher cumulative incidence among children aged 4 years compared with children aged 8 years was present for both sexes. The incidence curve for girls appeared to plateau at approximately 36 months, but cumulative incidence for boys continued to increase with age (Figure 3).

## Discussion

In 2016, a total of 15.6 per 1,000 (one in 64) children aged 4 years were identified with ASD at sites in the Early ADDM Network. Like previous years, considerable variability was reported across sites. The 2016 estimate for children aged 4 years was similar to the 2016 National Survey of Children’s Health (NSCH) weighted prevalence estimate of parent-reported ASD among children aged 3–5 years, 19.7 per 1,000 (*19*). The overall prevalence of 15.6 per 1,000 children aged 4 years in 2016 was higher than the 2014 estimate for children aged 4 years using DSM-5 criteria, 14.1 per 1,000 children (*11*). In addition, the cumulative incidence of ASD diagnoses at age 48 months was higher for children born in 2012 than for children born in 2008, which indicates a higher rate of diagnosis for the younger cohort.

The pattern of higher prevalence among males has been reported regularly in studies of ASD (*20*). Prevalence of ASD among boys was 3.5 times higher than among girls, the same as the adjusted prevalence ratio estimate from NSCH. The ADDM Network estimate among children aged 8 years of 4.3 boys to each girl identified with autism in 2016 was higher than the estimate for children aged 4 years. ASD sex ratios previously had been found to be lower among those with intellectual disability (*21*); therefore, a lower sex ratio could be expected among children aged 4 years, who are more likely to be identified with co-occurring intellectual disability. Cumulative incidence patterns also differed by sex, with a steady increase in diagnoses with age for boys but an apparent plateau for girls at approximately age 36 months.

In 2016, the ASD prevalence among white and black children aged 4 or 8 years did not differ within each age group. At one site (New Jersey), ASD prevalence among Hispanic children aged 4 years (32.1 per 1,000 children) was higher than among white and black children aged 4 years, the reverse of the pattern among children aged 8 years (*8*). The overall prevalence for Hispanic children aged 4 years at other sites was 11.0 per 1,000 children. Continued monitoring of trends among racial and ethnic groups is needed to assess disparities in ASD identification.

### Co-Occurring Intellectual Disability

Among the three sites (Arizona, New Jersey, and North Carolina) with intellectual disability data for 2014 and 2016, data were available for higher percentages of children aged 4 years in 2016 compared with 2014 (*11*). An increase in the percentage of children aged 4 years with ASD and co-occurring intellectual disability was reported at two of the three sites (New Jersey [from 45% in 2014 to 53% in 2016] and North Carolina [from 45% in 2014 to 66% in 2016]). These increases persisted when DSM-5 case classification was used for 2014 data, suggesting the increase in co-occurring intellectual disability was not driven by differences in diagnostic criteria.

In 2016, a lower percentage of children aged 8 years with data about intellectual disability had co-occurring intellectual disability (33%) (*8*) compared with children aged 4 years (53%). Among children aged 8 years, more girls than boys had co-occurring intellectual disability, whereas no difference was found in presence of co-occurring intellectual disability between sexes for children aged 4 years, consistent with previous reports. Median age at earliest evaluation and ASD diagnosis was similar for children aged 4 years with and without intellectual disability; however, median age at earliest evaluation for children with data about intellectual disability was lower than for those who did not have intellectual disability data available. In contrast, children aged 8 years with ASD and intellectual disability were more likely to be evaluated and receive an ASD diagnosis at a younger age (*8*). Differences in intellectual disability findings between the two age groups are difficult to interpret because of differences in age group characteristics and reasons for intellectual testing.

###  Age at Earliest Evaluation and Diagnosis

Overall, the percentage of children aged 4 years with ASD who had an earliest evaluation at age ≤36 months increased from 74% in 2014 (*11*) to 84% in 2016. This is progress toward the *Healthy People 2020* goal of increasing the percentage of children with ASD who receive their first developmental evaluation by age 36 months. The percentage of children aged 4 years with documented ASD diagnoses increased from 58% in 2014 to 71% in 2016. Median age at earliest diagnosis among sites was approximately the same range in 2014 (28–36 months) and 2016 (29–36 months).

Progress in early identification of ASD over time and between groups is difficult to ascertain on the basis of median age at diagnosis alone because the total prevalence of children with an ASD diagnosis varies among sites and by age. Cumulative incidence is a more useful approach to comparing patterns. With this approach, an overall increase in cumulative incidence of ASD diagnosis was apparent among the cohort of children aged 4 years compared with those aged 8 years in the same area, starting at age 26 months and continuing through age 48 months. If the pattern continues through age 8 years, a higher prevalence among children aged 8 years might be expected during ADDM Network surveillance year 2020.

By age 48 months, the pattern of higher cumulative incidence among children aged 4 years compared with children aged 8 years was present for the majority of sites. Cumulative incidence was approximately the same between age groups in Arizona and was higher for children aged 8 years than for those aged 4 years in North Carolina. The pattern in Arizona is consistent with the relative stability over time of prevalence estimates for children aged 8 years for that state. The pattern in North Carolina could be in part because education records in one surveillance-area county were only available for children aged 8 years.

## Future Directions

Monitoring trends in cumulative incidence of ASD can provide greater insight into overall increases in prevalence and progress toward the goal of increased early identification. Cumulative incidence of diagnoses also affords greater comparability to ADDM Network data for children aged 8 years than the median age at ASD diagnosis because of the difference in length of follow-up. Further study is needed of trends in prevalence among racial and ethnic groups as well as how sex and intellectual disability status might affect these patterns. Children identified by the ADDM Network are receiving evaluations that could lead to receipt of services whether they have a documented ASD diagnosis or not; information could be collected in the future to describe when children begin services or what types of services typically are used.

## Limitations

The findings in this report are subject to at least three limitations. First, ADDM Network surveillance methods depend on availability, quality, and completeness of records reviewed by each site. Education and early intervention records are not available for review in all sites, which could result in incomplete ascertainment. Second, the same surveillance case definition for intellectual disability was used for children aged 4 years as for children aged 8 years; however, children aged 4 years might not be given a clinical diagnosis of intellectual disability because of lack of stability of IQ at younger ages. Finally, ADDM Network sites are funded through a competitive process, and surveillance area populations are subsets of state populations that might not be representative of the whole state or entire United States. Racial and ethnic compositions vary across site populations and could contribute to variability in findings.

## Conclusion

Data from surveillance year 2016 suggest ASD prevalence was higher among children aged 4 years compared with 2014, although wide variability in estimates could reflect variable success in improving community identification. In addition, these children aged 4 years (born in 2012) had a higher cumulative incidence of ASD diagnoses by age 48 months compared with children aged 8 years (born in 2008), which indicates more early identification of ASD in the younger group. However, the overall median age at earliest diagnosis was 33 months, well above the youngest age at which ASD can be identified (*13*). Although progress has been made to evaluate more children by age 36 months, work remains to improve early diagnosis so children can receive timely services.
